# Spinal Wound—Tetanus—Death and Post Mortem

**Published:** 1879-07-20

**Authors:** A. B. Tadlock

**Affiliations:** Tennessee


### Southern Medical Record.

EDITORS:

T. S. POWELL, M.D.   W. T. GOLDSMITH, M.D.   R. C. WORD, M.D.

*R. C. WORD, M.D., Managing Editor.*

☞All Communications and Letters on Business connected with the RECORD must be addressed to the Managing Editor.

VOL. IX.   ATLANTA, GA., JULY 20, 1879.   No. 6.7

### ORIGINAL AND SELECTED ARTICLES.

### *SPINAL WOUND—TETANUS—DEATH AND POST MORTEM.*

Reported to the Knox County, Tennessee, Medical Society, in Regular Session, May 18th, 1879.

BY A. B. TADLOCK, A.M. M.D., OF TENNESSEE.

April 15th, 1879. Was summoned by Dr. Failing with instructions to meet him, ten miles in the country, with the necessary instruments for extracting, if possible, a piece of knife-blade 1½ inches long, from the shoulder-blade of a young man.

Two brothers had fought. Amos, the younger, had stabbed Andrew, who lay on the bed, a little nervous and excited at my arrival, but not suffering. This was on Monday, October 18th, nearly three days after the affair had taken place.

It had been a quarrel over bridling a horse; Amos cut the bridle rein and was rebuked by Andrew. Amos then rushed at him with the knife; in trying to run Andrew slipped and fell on his hands and knees, in which position he received two ugly stabs, one in the inferior angle of the left scapula, the other, one inch to the left of the seventh dorsal vertebra. A large pocket-knife was exhibited with a considerable portion of the large blade broken off, and supposed to be in the wound of the scapula.

The probe did not elicit a metallic impression as had been interpreted by the doctor upon his first examining the wound, nor could any satisfactory results be obtained by the most careful examination of the wound near the spine. The patient was put under the influence of chloroform and ether, and the scapular wound enlarged so as to see plainly where the knife had penetrated that bone. Thinking the blade might have become fixed in a rib beneath, and there broken off, I trephined, but found nothing.

The other wound, by its uneven edges, had a more suspicious appearance; the opening was large enough to admit the index finger easily up to the second joint, and with it I made a thorough search, as also did Dr. Failing. Probing, which had failed in both our hands previously, afforded now no better results while the patient was under an anesthetic.

From this and the behavior of the boy Amos when questionned, it was suspicioned that he had broken the knife purposely after wounding his brother, for if the blade had penetrated, as the position of the wound indicated, the articular region of the ribs to the depth of one and a half inches (the approximate length of the missing fragment), beyond the depth of our probing, it must have entered the chest, whereas we had no constitutional symptoms warranting any such inference; and if otherwise it could have possibly entered the spinal canal, there would be paralysis, which was not the case. Therefore further procedure for the present was abandoned, and cold water dressing, with laxatives and dietetic treatment, ordered.

I did not further visit the patient, but Dr. Failing kept faithful watch, and I was informed, almost daily, of the patient's condition, until he was considered almost out of danger. The scapular wound healed almost by first intention, and on the tenth day after the operation, having had no untoward symptoms, the patient dressed himself and sat up in a chair; appetite and digestion good; very little discomfort had been experienced night or day. He was a healthy-looking young man, but was said to have had for several years a chronic cough.

But here I will introduce the notes of the case, reported by Dr. Jno. W. Failing, the attending physician.

*Case*, A. J. M., age 18. Primary casualty: Knife wounds or stabs in back, 1 to 2 o'clock, p.m. April 11th, 1879.

Secondary effect: Traumatic trismus.

Result: Death from convulsions, 9 o'clock a.m., April 26th. 1879.

Notes.—April 11th, 2 p.m. Found patient with two stabs of knife; one over the centre of the inferior part of the left scapula, superficial length ¾ inch; depth, penetrating the scapular bone; direction, perpendicular to the surface; hemorrhage, slight.

The other stab, an inch to the left and slightly below the sixth dorsal vertebra, about the same length, superficially, as the first, with an irregularity or nich apparently caused by a twist of the knife in withdrawing it; direction, anteriorly, slightly downwards and to the right; hemorrhage severe and very obstinate.

I was informed that 1¼ to 1½ inches of the knife-blade was broken off, and supposed to be in one of the wounds, but was unable to find it; patient was sinking rapidly from shock and hemorrhage; pulse, from 40 to 45.

Checked hemorrhage and endeavored to rally patient, and dressed wounds with cold water. Patient passed a sleepless night; pulse rising to 70.

April 12th. Patient rallied considerably by morning; pulse 80, but thready; had passed no urine. Gave cathartic pill, comp., one every 4 to 6 hours, and decoc. scoparii. Passed urine at 12 m.; rested easier during remainder of day and night; pulse fuller and regular.

April 13th. Continued restless with but little sleep; looks anxious; pulse fitful; bowels not yet moved. Gave castor oil and clyster of oil and turpentine.

April 14th. Bowels moved early; patient seems better in body and mind; pulse full and strong. At 11 a.m. Dr. A. B. Tadlock, of Knoxville, made a careful search for the knife blade, without success. Patient seemed to rest easy after the examination, and with a dover's powder passed a quiet night.

April 15th. Doing very well, but a little feverish. Gave cathartic pills to move bowels.

April 16th. Bowels moved very freely in the morning; continuing to move too frequently, gave pill opii, and ordered dover's powders at night.

April 18th. Patient seemed to be doing exceedingly well; wounds healthy; pulse good; mind lively; rest but little interrupted; nerves quiet; bowels regular; evacuations healthy; appetite good; no pain. Continued water dressing, light diet and no medicines, except a continuation of chlorate of potash, to relieve a cough which had been troubling him all winter, and now somewhat strained the wounded muscles of his back and shoulder.

April 20th. Skin of shoulder somewhat excoriated. Discontinued water dressings, and applied cerate simp. Patient continued improving until the 22d. As he did not seem to require my attention, did not see him again until the evening of the 24th, when I found that his bowels had not been moved since 22d. Patient complains of oppression and slight cramping or griping of his bowels. Gave ½ oz. senna in decoction.

April 25th. Bowels moved at 9 or 10 a.m. He was still suffering griping or cramp. Sent dover's powders to be given at once. Saw him myself at 12 m.; he had been unable to swallow the dover's powders. Patient looked haggard and anxious; pulse uneven; eyes staring and complained of cramps and severe pain at the pit of the stomach; spasmodic contraction of the diaphragm and muscles of the upper abdominal region; spasms short in duration, occurring in groups of from one to half a dozen within a space of one or two minutes, with intervals of five to fifteen between the groups. These were induced in an aggravated form by attempting to swallow. I injected subcutaneously ¼ gr. morph. sulph. over the umbilical region, repeating it at intervals of from one to two hours until upwards of a grain was used; also gave 30 gr. brom. potass. Pain somewhat abated; spasms less severe for short time after each injection, but more violent in 2 or 3 hours. Shortly after midnight a strong convulsion occurred, preceded by one or two slight ones. Gave morphia as before, and as soon as any effect was shown administered gr. xxx chloral hydr. between the spasms.

April 26th. About 2 a.m. patient fell into a sleep disturbed by slight spasms. 4 a.m. Awoke in a strong convulsion. Again used morphia and endeavored to give chloral, but failed, as the spasm of the throat threatened suffocation. The muscles of the jaws showed an increase of rigidity during the last 10 or 12 hours, but not much spasmodic contraction, and were never completely set.

From 4 o'clock until 9 a.m., when he died, he grew steadily worse, the spasms becoming more frequent and somewhat increased in severity, about the diaphragm, chest and throat; pulse more frequent and feeble, reaching 150, and scarcely perceptible just after a convulsion.

Whether death resulted from dyspnœa, or spasm of the heart, I am unable to say, but think the former, from the fact that his pulse beat some seconds after his breathing ceased.

A post mortem was held by Dr. A. B. Tadlock, Dr. J. Park and others, April 27. The knife blade being found to have penetrated the body of the 7th dorsal vertebra, impinging on and wounding the surface of the spinal cord.

*Sectio Cadaveris.*—(Notes taken by J. D. COLLIER, a Med. Student). Condition good; skin exsanguineous and tinged a little yellow. Eyes glassy and very flaccid, iris unusually so, though normal in size; the anterior chamber appeared as if the vitreous had replaced the aqueous humor. Back of ears and sides of neck thickly ecchymosed or suggillated; gums very anæmic and tongue coated; rigor mortis only noticed in the tightly clenched right hand; nails blue.

Wounds. Scapular nearly healed, and looking healthy, with a little laudable pus about the trephined opening. This wound is located near the lower angle of the left scapula. Wound one inch to the left of the tip of the spinous process of the 6th dorsal vertebra unhealed and presenting a mortified appearance which, upon opening, proved to be so, extending to the osseous structures, and affording exit for offensive gases from the thoracic cavity indicating an opening thereto. This, however, proved to have been the result of gangrene, and not of the original wound.

Failing, with considerable care and scrutiny, to find in this wound any foreign substance, the cadaver was turned over and the chest opened. The pectoral muscles were as highly tinted as if they had been treated with ammonia. Extensive pleuritic adhesion on the left side, and gangrene beginning; lungs, spleen and pancreas, black and full to engorgement; heart flaccid, pretty well filled and livid; 1½ to 2 oz. sanguinolent; serous fluid in pericardium; stomach and bowels full of gas; 8 to 10 oz. fluid in left pleural cavity; left lung being partly hepatized; no sign of foreign substance in cavity or its walls from the inside; liver plethoric; all valves of heart completely collapsal; fibrous clot, structural in appearance and consistency, 2 inches long and twice the size of a large goose quill, was entangled in the carneous columns.

Not yet satisfied with our search for the foreign substance, we returned to re-examine the spinal wound, and, after dissecting away the entire muscular tissues, and laying bare the spines and lateral processes of the 6th, 7th, and 8th dorsal vertebra, and the articular extremities of the corresponding ribs, pus was seen to issue from the bone near the 7th spinous process. Bone-forceps and chisel were brought to our assistance, and after removing the spinous process and lamina, and displacing the cord, the fragment of a knife blade which had penetrated the meninges and a small lateral portion of the cord, was found fixed almost perpendicularly in the body of the 7th dorsal vertebra, and with much difficulty removed, being 1⅜ inches in length and 9-16th in width at the broken end, teminating in a point. The original knife was a large, heavy pocket knife, 6 ¾ inches (handle and blade) with a small and a large blade. Of the latter, 1¾ in. was left in the handle. By replacing the whole knife into the wound, and drawing up the integument, it was evident that the whole blade was buried beneath the parts with a force that depressed the skin to the depth of half an inch of the hilt or handle, in other words up close to the hand that held the knife.

Owing to the peculiar circumstances under which the post mortem was performed, further investigation was altogether impracticable.

This, as I conceive, extraordinary case, is peculiarly characteristic of the following observed facts relative to the causative influence of wounds upon the development of traumatic tetanus, viz: "The condition of the wound, its tendency to heal, gives no criterion by which to judge of the liability to the disease." "There is no limit to the time which may elapse between the injury and the outbreak of tetanus." (?)

It would be a rational inference to suppose that just such a wound with the retention of the foreign substance would be the most likely of all to produce the disease. Yet statistics show the smallest per cent. of tetanus originating from wounds of the trunk—smaller than that of any other region of the body.

In this connection the surgical volume, part 1st of the "Medical and Surgical History of the Rebellion" furnishes interesting information. Only 642 cases of injury to the vertebræ are therein reported, and only 2 of these were incised wounds. The similarity of one of these to our case induces me to make special mention of it.

Priv. —— Co. B, N. Y. Engineers, was stabbed in the back with a knife; was completely paraplegic from the injury; urine had to be drawn off, and croton oil in 3 gtt. doses only produced bowel dejections after three days. Sphacelus of lower extremities followed and extended rapidly to the spine of the sacrum. Died of exhaustion 36 days after receiving the wound.

Sectio cadaveris: "The 4th, 5th and part of the 6th dorsal vertebræ were sawn longitudinally to exhibit blade of the knife, which appears to have been broken off at the time of injury and remains fixed in the specimen," which may be seen No. 1160, in the Army Medical Museum at Washington, D. C. But, as shown in the wood cut, the knife did not enter the spinal canal.

While 55.5 per cent. of injured vertebræ proved fatal, tetanus only supervened in 7 cases out of all there reported; and of 73 cases in which the foreign substance either could not be found or was so impacted as to prevent its removal, 55 died; and 42 proved fatal out of fifty-four cases in which the spinal cord was involved—one survived 8 days after receiving a conoidal pistol ball into the spinal canal through the apophesis of the 8th dorsal vertebra, which passed upward through the medulla spinalis as far as the 1st cervical vertebra.

The spinal meningetis and disorganization of tissue, also the pyæmic feature of our case as evidencced in the disorganization of the blood, extensive sphacelation of wound, and internal organs, and the large semi-organized fibrinous clot found in the heart in post mortem, afford scope for argumentation as to cause and effect. I do not say, however, that it was pyæmic trismus, even if I could subscribe to that classification, nor that the pyæmia was at all sequential to the tetanus, but believe it only to have been concomitant with those dreadful peripheral vaso-motor commotions which drove the blood to the centres, and produced the resulting engorgements and congestions, inflammation and hepatization of lungs, with the adhesions.

I only have to add that, had the location of the knife-blade been known, trepannation of the vertebra would have been the only means by which removal could have been effected, and the experience and testimony of the most brilliant surgeons in history furnish no encouragement whatever for its probable success in our case.

In the discussion that followed the reading of the above paper, Dr. Carriger urged the importance of giving morphia for the effect, regardless of the doses, even if it required ½ gr. every 15 minutes to produce the desired impression.

Dr. Kennedy did not think the Doctor could have used any better agents than those he did make use of. Dr. Kennedy said: "In this case there are many points of interest, not only in the treatment of the physician, but in the boldness of the surgeon, although I do not think the boldness went far enough. I think it might be made a matter of future investigation in the Society. The paper that has been read is one of very great importance."

Moved that the paper and report be made the special order of the next meeting. Carried.

The author has to add that further boldness to have accomplished anything at all would have necessitated trephining the spine, which finds no encouragement in history even under more favorable circumstances.

Dr. Cline, June 16th, 1814, in St. Thomas Hospital, with trephine, saw, mallet and chisel, removed the arches of the 7th and 8th dorsal vertebræ in a man æt. 26, who had fallen, the day previous, and fractured the spinous process of these two, with that of the 9th, crushing them in upon the cord. The man lived 17 days, and, after his death, the operator "candidly admitted that the operation hastened his end." Tyrrell, Wickam and Attenburron severally repeated the operation with unfavorable results. Not daunted, however, other operators tried their hands, viz: Barton, Smith, Rogers, Holscher, Mayer, Langier, South, Edwards, Blair, Blackman, Potter, Stephen Smith, Hutchison, Goldsmith, Jones, McDonnell, Gordon, Tellanx, Felicet, Maunder, Willet and Tyrrell a second time. Pare, Heister, Sir A. Cooper and many of the older authors discussed the question with much acerbity.

More recently Brown Sequard strenuously advocated the operation on "physiological grounds." Then extended papers, by Felicet; Nunnally's Address; Discussions in the British Medical Societies, and Ashburst's Monograph, with analysis of 400 cases of spinal injuries, have not failed in furnishing evidence of thorough study and pretty reliable data, and after an examination of the whole subject with its almost boundless literature, Surgeon J. A. Lidell declares that he "has failed to find one completely successful case on record." Jobert characterizes the operation as "*barbare et ridicule*," and Malgaigne calls it a "desperate and blind one."

When there is positively no hope of saving life, experiment is justifiable, yea possibly science and humanity may demand the experiment, but where experimental knowledge has been so conclusively obtained as evinced by efforts so many times repeated, I should no longer consider the operation experimental, but one either to display presumed skill, or culpable ignorance on the part of the operalor.

Questions by grand jury :

"If the knife had been found could it have been extracted without producing death?"

"Think the effort to extract it would at least have made matters worse."

"If the blade had been extracted would he probably have recovered?"

"Not likely."

"Can you account for the patient getting apparently so nearly well, and then getting worse and dying?"

"Not positively. We might infer that after recovery from shock sufficient time is necessary for the progressive development of those nerve or other conditions growing out of wounds, or other causes which precede and introduce the final and fatal exhibition of diseased action."

